# Characterization of the complete chloroplast genome of *Ligusticum sinense*, as a Chinese herb to treat toothache in China

**DOI:** 10.1080/23802359.2020.1808103

**Published:** 2020-08-17

**Authors:** Qingwei Wu, Hui Wu, Li-Kang Wang, Xin Zhao

**Affiliations:** a Central South University Xiangya School of Stomatology, Changsha, Hunan, China; b Traditional Chinese Medicine Department, The Second People’s Hospital of Datong, Datong, Shanxi, China; c Department of Pain Management, The Third Medical Centre, Chinese PLA General Hospital, Beijing, China; d The Molecular Pathology Lab, The Second People’s Hospital of Datong, Datong, Shanxi, China

**Keywords:** *Ligusticum sinense*, phylogenetic analysis, chloroplast genome

## Abstract

*Ligusticum sinense* is a popular herb in Chinese medicine. The circular double-stranded complete chloroplast genome of *L. sinense* was 146,342 bp in length, exhibiting a typical quadripartite structure. It contained a large single-copy region (LSC) of 91,788 bp, a small single-copy region (SSC) of 17,618 bp and two identical inverted repeat (IR) regions of 18,468 bp each. The overall nucleotide composition of chloroplast genome sequence is: A (30.8%), T (31.6%), C (19.2%), G (19.4%) and the total G + C content of 38.6%. The chloroplast genome contained 127 genes, including 83 protein-coding genes, 36 transfer RNA genes and 8 ribosomal RNA genes were annotated. The total of 15 genes duplicated in one of the IR, including 6 tRNA, 4 rRNA, and 5 protein-coding genes. The ML phylogenetic tree indicated that *L. sinense* is closely related to *L. tenuissimum* in the phylogenetic relationship.

*Ligusticum sinense* belongs to the Umbelliferae family that has been used to relieve toothache and treat other oral diseases as one of Chinese medicine in China (Wei et al. 2014). In China, its Chinese named Chuan-Xiong as the herb that was also used to flavor food and add fragrance to cosmetics because of its warm and spicy qualities (Wang et al. 2011). At present, the study of *L. sinense* is only on the chemical composition and the reports on genomics and molecular biology are rare, which affects the further research and scientific utilization of this species. In this study, we published the chloroplast genome of *L. sinense*, which can help to research the phylogenetic relationship information, also can be useful for traditional Chinese medicine utilization research for future.

The samples of *L. sinense* were obtained from the herb market near the Hunan University of Chinese Medicine (47.28 N, 112.90E) located at Changsha, Hunan, China, and also preserved in liquid nitrogen for further study. The total genomic DNA of *L. sinense* was stored in Hunan University of Chinese Medicine (No. HNUCM-01). Total genomic DNA of *L. sinense* was isolated using the Plant Tissues Genomic DNA Extraction Kit (TIANGEN Biotech., Beijing and China) and sequenced. Adapters and low-quality reads were removed and controlled using FastQC (Andrews 2015). The chloroplast genome of *L. sinense* was assembled using MitoZ (Meng et al. 2019) and annotated by Geneious 8.1.7 (Kearse et al. 2012). The genes in chloroplast genome were predicted using CPGAVAS (Liu et al. 2012) and corrected using DOGMA (Wyman et al. 2004).

The circular double-stranded complete chloroplast genome of *L. sinense* (GenBank Accession number: NK9214541) was 146,342 bp in length, exhibiting a typical quadripartite structure. It contained a large single-copy region (LSC) of 91,788 bp, a small single-copy region (SSC) of 17,618 bp, and two identical inverted repeat (IR) regions of 18,468 bp each. The overall nucleotide composition of chloroplast genome sequence is: A (30.8%), T (31.6%), C (19.2%), G (19.4%) and the total G + C content of 38.6%. The chloroplast genome contained 127 genes, including 83 protein-coding genes, 36 transfer RNA genes, and 8 ribosomal RNA genes were annotated. The total of 15 genes duplicated in one of the IR, including 6 tRNA, 4 rRNA, and 5 protein-coding genes.

To confirm the phylogenetic relationship of *L. sinense*, 16 plant species complete chloroplast genome sequences were aligned using MAFFT (Katoh and Standley 2013) and maximum-likelihood (ML) analysis was conducted using MEGA X (Kumar et al. 2018) with 2000 bootstraps values at all the nodes under the substitution model. The phylogenetic tree was drawn and edited using MEGA X (Kumar et al. 2018). The ML phylogenetic tree (Figure 1) indicated that *L. sinense* is closely related to *L. tenuissimum* in the phylogenetic relationship (Figure 1). This complete chloroplast genome of *L. sinense* can provide useful information for phylogenetic studies and will be useful for traditional Chinese medicine utilization research for future.

**Figure 1.**
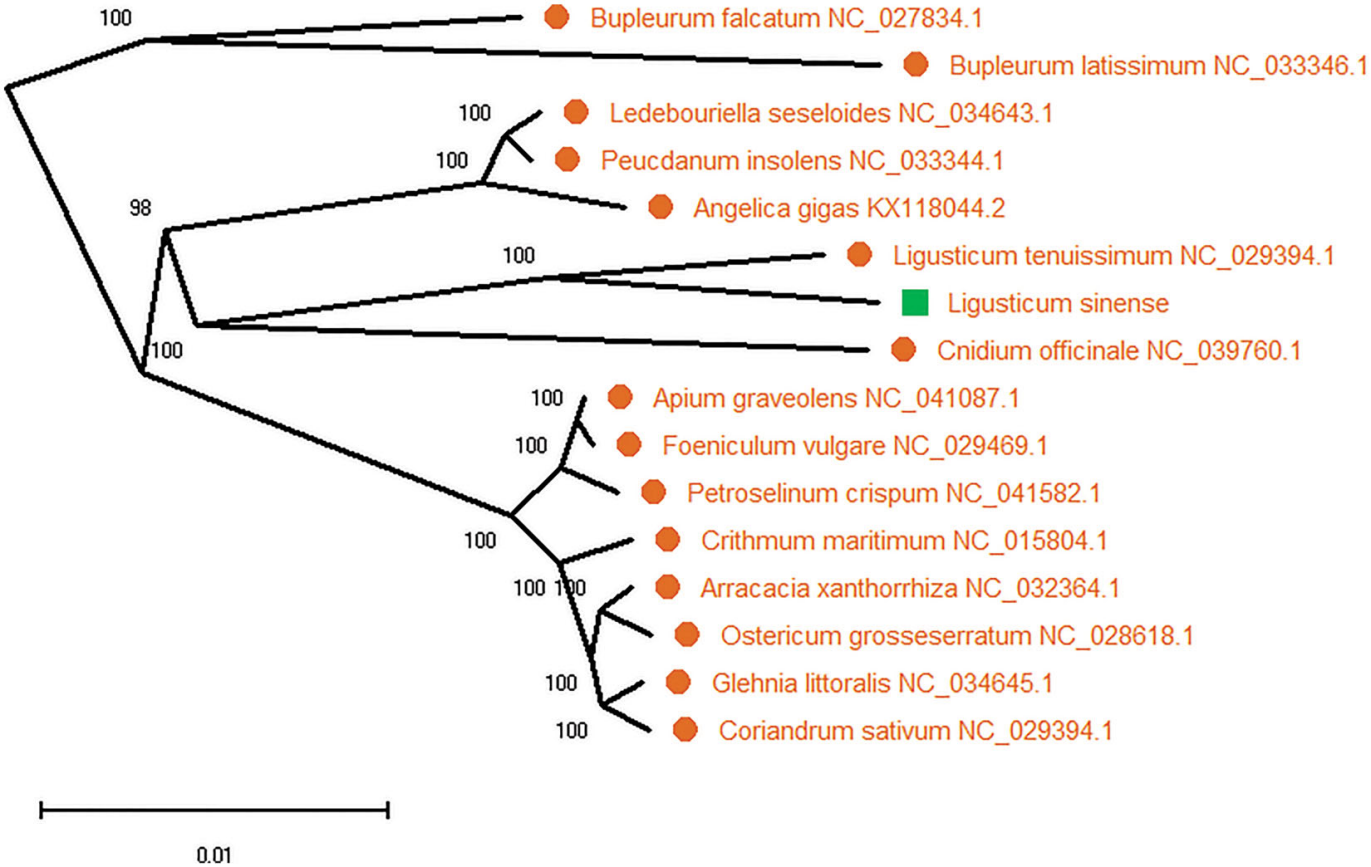
Phylogenetic maximum likelihood tree with *L. sinense* based on 16 plant species complete chloroplast genomes. The number on each node indicates bootstrap support value from 2000 replicates.

## Data Availability

The data that support the findings of this study are available from the corresponding author, upon reasonable request. The data that support the findings of this study are openly available in *Ligusticum sinense* at NCBI, reference number [reference number NK9214541].
